# MRI evaluation of left ventricular remodeling following stent angioplasty of native coarctation

**DOI:** 10.1186/1532-429X-13-S1-P188

**Published:** 2011-02-02

**Authors:** Alicia Lim, Hannah R Bellsham-Revell, Thomas Krasemann, Eric Rosenthal, Shakeel Qureshi, Gerald Greil, Philipp Beerbaum, Reza Razavi, Aaron Bell

**Affiliations:** 1 King's College London, London, UK; 2 Guy's and St Thomas' Foundation NHS Trust, London, UK

## Aim

To assess left ventricular remodeling after stent angioplasty for native coarctation using serial cardiac MRI.

## Introduction

Coarctation of the aorta in the older child and adult can be successfully treated by percutaneous stent implantation. Although afterload will be reduced by coarctation stenting, other factors including systemic blood pressure will influence left ventricular remodeling.

## Methods

Ethical and institutional approval was obtained. The internal cardiac database was searched for patients who had stenting for native coarctation and had MRI scans performed pre and post intervention.

## Results

**Figure 1:**
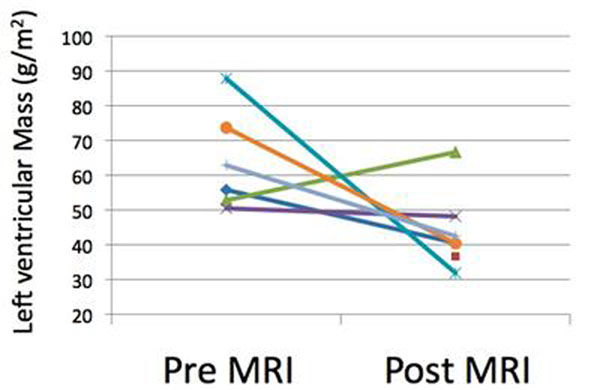


**Figure 2:**
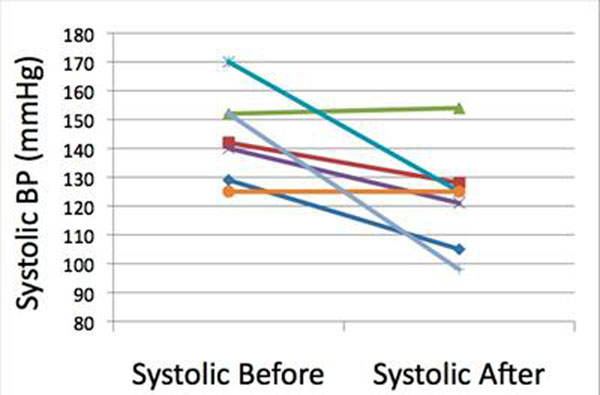


7 patients were identified that fell into this group. Median (range) age at stent implantation was 14.9 (9.1-27.1) years and MRIs were performed 0.2 (0.09-0.9) years after implantation. There was no change in end diastolic volume, with a small decrease in end systolic volume 29.4mls/m^2^ (17.9-32.0) to 20.2mls/m^2^ (16.2-25) causing a slightly increased cardiac output 3.5l/min/m^2^ (2.8-4.4) to 4.1l/min/m^2^ (2.9-4.6). Left ventricular mass decreased from 59.33g/m^2^ (50.46-87.80) to 40.54g/m^2^ (31.89-66.64), although there was one patient in whom LV mass increased. Figure 1 demonstrates the changes in ventricular mass and figure2 the changes in systolic blood pressure. In the one patient in where mass did not fall despite adequate relief of coarctation there was no change in systolic blood pressure.

## Conclusions

We demonstrate that MRI at a relatively early stage after coarctation stenting can show evidence of left ventricular remodeling when there is a concomitant fall in systemic blood pressure.

